# Fault diagnosis of rotating machinery under imbalanced samples based on outlier-removal HCAB-SMOTE-LOF

**DOI:** 10.1016/j.isci.2026.117955

**Published:** 2026-10-26

**Authors:** Zhiqiang Liao, Zhijia Yan, Baozhu Jia, Xuewei Song

**Affiliations:** 1 Naval Architecture and Shipping College, Guangdong Ocean University, Zhanjiang, China; 2 Technical Research Center for Ship Intelligence and Safety Engineering of Guangdong Province, Zhanjiang, China; 3 Guangdong Provincial Key Laboratory of Intelligent Equipment for South China Sea Marine Ranching, Zhanjiang, China

**Keywords:** rotating machinery, fault diagnosis, HCAB-SMOTE-LOF, imbalanced samples

## Abstract

In rotating machinery fault diagnosis, the original feature space often suffers from anomalous outliers, limited sample availability, and imbalanced class distributions. These issues significantly impede the accurate recognition of minority-class samples and lead to suboptimal diagnostic efficiency. To tackle these challenges, this paper proposes a hybrid method of HCAB-SMOTE-LOF for diagnosing imbalanced fault diagnosis under outlier contamination. The core idea is not a simple combination of Local Outlier Factor (LOF) and Hybrid Clustering Affinity Synthesis Minority oversampling technique (HCAB-SMOTE), but a coupling mechanism in which LOF-based majority-class purification constrains the boundary region of HCAB-SMOTE, so that synthetic samples are generated in reliable minority-class clusters. To extract the fault-relevant time-frequency features, high-pass filtering is applied to the raw vibration signals to remove low-frequency components. The LOF is introduced to eliminate samples that are clearly abnormal or mislabeled in the feature space. On this basis, the HCAB-SMOTE algorithm is employed to synthetically generate minority-class fault samples, thereby improving the quality and distributional plausibility of the generated samples and alleviating the adverse impact of data imbalance on classifier performance. The effectiveness of the proposed method is validated using both a laboratory dataset and the Ottawa motor dataset. Furthermore, a comparative analysis is conducted between the proposed method and several oversampling strategies applied during data processing, including the use of original data, SMOTE, Borderline-SMOTE, and HCAB-SMOTE. Experimental results demonstrate that the proposed method consistently outperforms all existing oversampling methods across all evaluation metrics, confirming its marked advantages in outlier removal and sample-distribution optimization and thus its capability to effectively detect rotating machinery faults.

## Highlights

• An outlier-aware adaptive oversampling framework is developed for bearing fault diagnosis
• LOF purification is coupled with HCAB-SMOTE to constrain synthetic sample generation
• The coupling avoids outlier amplification and preserves informative boundary samples
• Bagging ensemble ensures robust diagnosis under 1:7 and 1:10 imbalance ratios

## Graphical abstract

**Figure:**
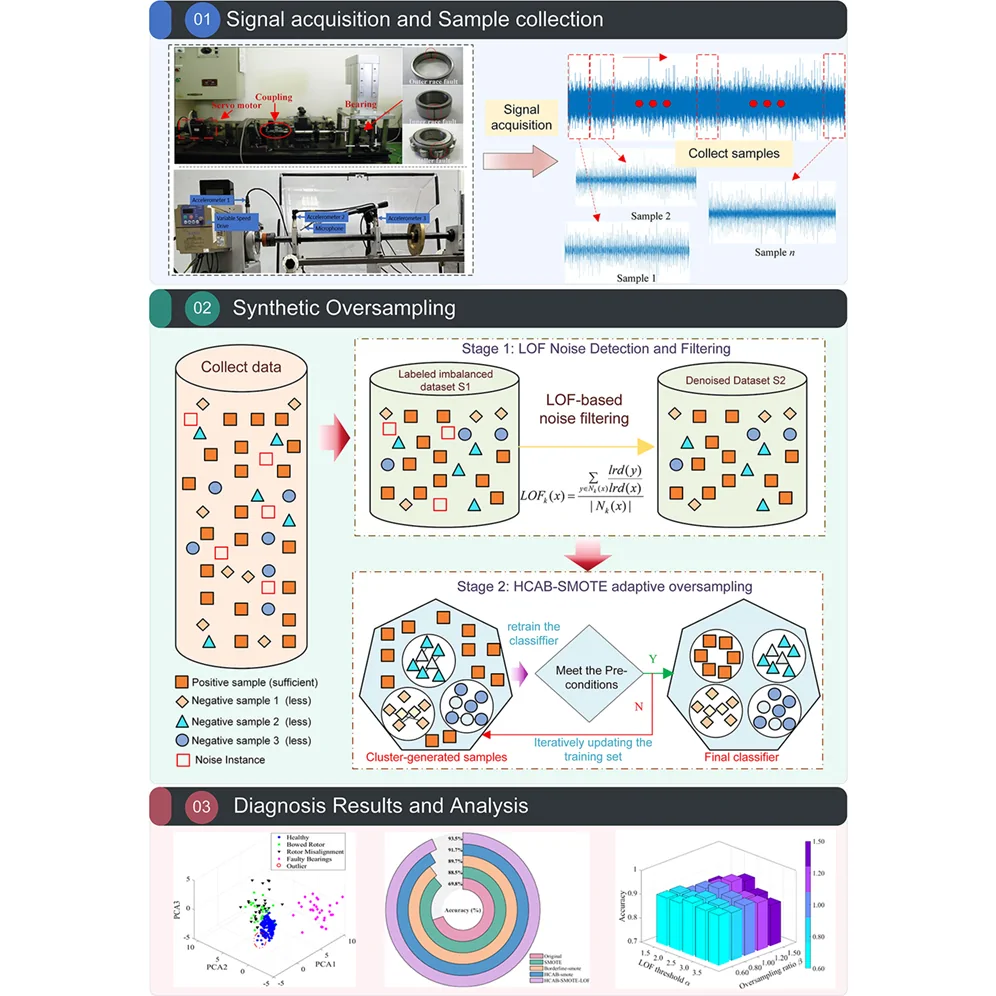

## Teaser

Applied sciences; Network

## Introduction

Rotating machinery serves as a core component in industrial systems and is widely employed in sectors such as energy, transportation, and manufacturing, where it undertakes the critical tasks of power transmission and energy conversion. Bearings, as the key supporting elements of rotating machinery, have a health condition that directly affects the operational reliability, safety, and service life of the entire system. Once a bearing fault occurs, it may lead to equipment shutdown, production line interruption, or even severe safety accidents. Therefore, research on fault diagnosis for bearings in rotating machinery is of significant engineering value and practical importance. However, in real industrial scenarios, rolling bearings operate under normal conditions for the vast majority of their service life, making it difficult to acquire a large number of fault samples. As a result, the available fault samples for model training are extremely limited. This small-sample issue prevents data-driven diagnostic models from adequately learning fault characteristics, thereby increasing the risk of overfitting or model bias and severely constraining the accuracy and generalization capability of fault diagnosis techniques.

To address the issue of class imbalance in fault diagnosis, researchers have proposed a variety of improved methods from multiple perspectives, including data resampling,^1^ generative adversarial networks,^2^ hybrid learning,^3^ and outlier detection,^4^ thus forming a diversified development trajectory from traditional oversampling to generative models, and then to hybrid strategies and data quality assurance. Among these, the Synthetic Minority Oversampling Technique (SMOTE) has attracted widespread attention due to its simplicity and effectiveness. By generating new samples through linear interpolation between existing minority-class instances, SMOTE effectively mitigates the classification bias caused by data imbalance and significantly enhances the recognition capability for minority fault patterns.^5^^,^^6^^,^^7^ Al Majzoub et al.^8^ proposed HCAB-SMOTE, which first identifies borderline dangerous samples, then applies k-means clustering to partition the boundary regions and remove majority-class boundary noise, and finally performs oversampling on the effective clusters. This approach reduces the number of generated samples while simultaneously improving classification accuracy. Wang et al.^9^ developed CND-SMOTE, which comprehensively considers the distribution of samples from various classes within the neighborhood, thereby effectively increasing the fault diagnosis accuracy. Duan et al.^10^ introduced MeanRadius-SMOTE, which computes the geometric center and average radius of minority-class samples, so that the generation distances of new samples follow a normal distribution, effectively avoiding the production of useless or noisy samples. Wang et al.^11^ proposed the BDOP-GMM-SMOTE oversampling method based on Gaussian mixture models and boundary connectivity optimization, aiming to address the poor quality of generated data in classical SMOTE. However, although these methods are easy to implement, the quality of the generated samples heavily depends on the nearest-neighbor selection strategy. Moreover, they struggle to effectively remove outliers from the original data and are prone to generating noisy samples in boundary regions, which ultimately leads to blurred classification boundaries.

With the continuous advancement of deep learning, the introduction of Generative Adversarial Networks (GANs) and their variants has provided new perspectives for imbalanced learning. Through the adversarial game between a generator and a discriminator, GANs are capable of automatically learning the underlying distribution of original data, thereby generating high-fidelity synthetic samples. Their variants, such as WGAN and conditional GAN, further improve training stability and the controllability of generated samples by refining loss functions or incorporating auxiliary information. Compared with traditional oversampling methods, GANs can capture more complex multimodal distributions and have demonstrated superior sample generation capability in fault diagnosis for rotating machinery components such as bearings and gears, particularly in scenarios with diverse fault patterns. Yin et al.^12^ proposed a Variant Auxiliary Classifier Wasserstein GAN (VACWGAN) to address the scarcity of aero-engine bearing fault samples, introducing the Wasserstein distance and an auxiliary classifier to enhance the diversity of generated samples. Ruan et al.^13^ developed a collaborative optimization method combining CNN and GAN to tackle the performance degradation of bearing fault diagnosis under imbalanced datasets. However, the training process of GANs is highly sensitive to network architecture and hyperparameters, and with limited samples, they are prone to mode collapse or overfitting, which to some extent restricts their reliability for direct application to small-sample industrial data.

Furthermore, researchers have incorporated clustering and semi-supervised learning into imbalanced diagnosis, giving rise to hybrid approaches. These methods first employ clustering algorithms to partition the distribution of original samples, thereby identifying core regions and boundary regions. On this basis, they combine semi-supervised learning frameworks to fully exploit the latent information contained in a large number of unlabeled samples, which in turn assists oversampling or feature enhancement. Hybrid strategies are capable of simultaneously addressing data-level augmentation and feature-level discriminability improvement, making them practically valuable in industrial scenarios where labeling costs are high. Li et al.^14^ proposed a CJBM method based on gamma-jump density peak clustering (γDPC) and SMOTE, which adaptively determines the cutoff distance and cluster centers, and combines SMOTE oversampling with a LightGBM classifier, achieving excellent performance on semi-supervised and imbalanced bearing data. Kim et al.^15^ presented an interpretable fault diagnosis method integrating the short-time Fourier transform and Grad-CAM, which extracts critical frequency regions from spectrograms and computes anomaly point ratios, enabling reliable assessment of bearing health conditions. Wang et al.^16^ addressed the data imbalance problem in transformer fault diagnosis by employing SMOTE oversampling, LightGBM feature selection, and the Northern Goshawk Optimization algorithm to optimize the GBDT model, significantly improving the diagnostic accuracy and Kappa coefficient for minority fault classes. Nevertheless, the clustering process itself is sensitive to outliers, and semi-supervised training often involves multiple iterations, leading to a marked increase in overall computational cost. Moreover, the identification of anomalous samples remains insufficient, making it difficult to balance efficiency and accuracy in practical deployment.

In response to the limitations of the above methods in dealing with imbalanced data containing outliers, recent studies have empirically revealed the negative impact of outliers on the oversampling process. Kang et al.^17^ found through experimental analysis that directly applying SMOTE for oversampling in class-imbalanced datasets, if a few class samples contain outliers, not only cannot improve model performance, but also leads to a decrease in classification accuracy. The fundamental reason is that the linear interpolation mechanism of SMOTE relies on the nearest neighbor relationships between minority class samples, and outliers, as isolated points far from the cluster center, significantly distort the nearest neighbor selection strategy, causing the synthesized samples to deviate from the true fault feature distribution.^18^ To alleviate this problem, some researchers have attempted to combine outlier detection with oversampling methods. Among them, methods based on Local Outlier Factor (LOF) have attracted attention due to their advantages in identifying local density differences. The SMOTE-LOF method introduces the LOF algorithm before oversampling to screen for outliers in minority class samples, remove samples with high outlier factors, and then perform SMOTE generation.^19^ Experiments show that this method can effectively reduce the interference of outliers on the quality of synthesized samples and improve the generalization performance of the classifier. The study by Xu et al.^20^ further validated the effectiveness of embedding density-based anomaly detection in the oversampling process. By quantifying the degree of local outliers in the samples, it is possible to more accurately identify noisy samples in the boundary region, thereby avoiding the generation of invalid or misleading composite data at inter-class boundaries.

Although the aforementioned methods can alleviate class imbalance to a certain extent, actual rotating machinery fault data are inevitably contaminated by anomalous outliers due to varying operating conditions and strong background noise. These outliers can distort the nearest neighbor selection strategy in oversampling methods or interfere with the discriminative gradients of generative models, causing the synthesized samples to deviate from the physical characteristics of real faults. Consequently, in scenarios where minority samples are contaminated by noise, using outlier detection as a pre-processing step for oversampling is an effective way to ensure the quality of synthesized samples and improve the accuracy of unbalanced fault diagnosis. To address this issue, this paper further proposes a method that combines the Hybrid Clustered Affinitive Borderline Synthetic Minority Over-sampling Technique with the Local Outlier Factor (HCAB-SMOTE-LOF). Prior to generating synthetic samples via HCAB-SMOTE, LOF is employed to perform anomaly detection on the samples, identifying and removing those with high outlier factors. This effectively eliminates the interference caused by outliers on the classifier. Through this improvement, the distribution of the synthesized samples more closely approximates that of the true minority-class samples, significantly enhancing both the quality and authenticity of the generated data. Subsequently, a Bagging ensemble classifier is adopted, and performance is evaluated using metrics such as precision, recall, F1-score, and G-mean to achieve accurate fault identification for rotating machinery bearings.

The main contributions of this paper are as follows.

(1) This paper identifies and formulates the problem of co-occurring class imbalance and outlier contamination in rotating machinery fault diagnosis. It reveals that directly applying SMOTE series methods to outlier-contaminated data amplifies abnormal patterns and distorts the minority-class distribution, because outliers distort the nearest-neighbor selection on which linear interpolation relies.
(2) A coupled outlier-aware oversampling mechanism is proposed, in which LOF is embedded into the boundary-seed construction and sample-budget control of HCAB-SMOTE rather than being applied as an independent preprocessing step. Specifically, the boundary seed set is redefined on the LOF-purified distribution, and the LOF-based removal is jointly constrained by a 3% deletion cap and a 97% minority-retention requirement. This coupling prevents both outlier amplification during synthesis and over-cleaning of informative boundary samples.
(3) Provided systematic empirical evidence to demonstrate the effectiveness of the proposed framework. Through outlier visualization, comparative experiments, and ablation experiments, we found that this improvement mainly stems from the complementary effect between outlier detection and adaptive oversampling methods, rather than relying solely on the independent effects of these two methods.

## Results

### Fault diagnostic procedure

In practical engineering scenarios of fault diagnosis for rotating machinery bearings, the acquired vibration signals are generally contaminated by outliers due to complex operating conditions, environmental interference, and limitations of sensor acquisition. Moreover, the sample distribution across different health states is highly imbalanced, which tends to bias intelligent diagnostic models toward majority classes while resulting in insufficient recognition accuracy for minority fault types. These issues severely restrict the engineering applicability of diagnostic methods. To address these challenges, this paper proposes an intelligent diagnostic method for imbalanced fault samples in rotating machinery bearings, as illustrated in Figure 1. The overall procedure is described as follows.

**Figure 1:**
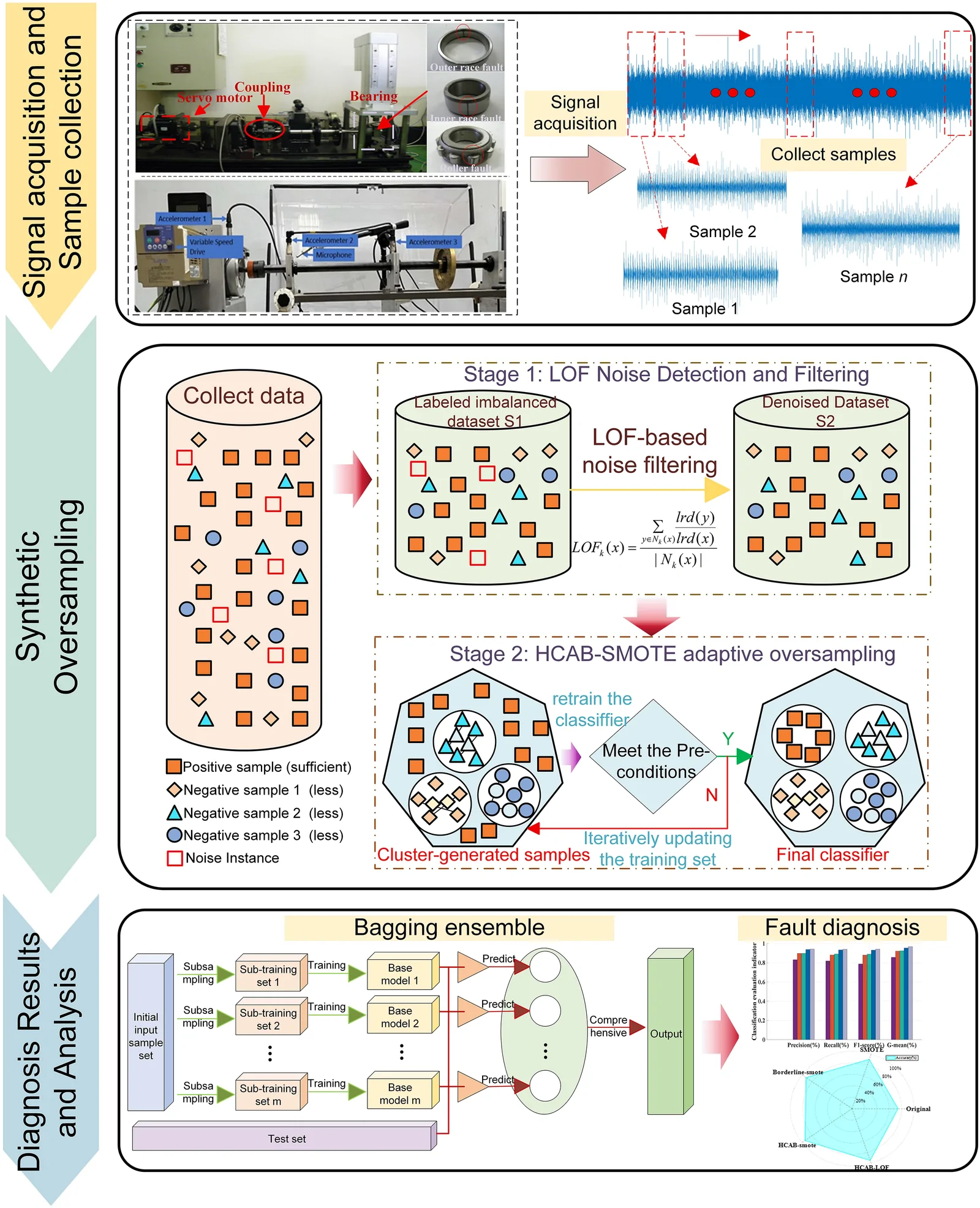
Theoretical framework of the proposed method

First, raw vibration signals are collected under various operating conditions and different fault types using a bearing fault test rig. The time-series signals are then segmented into samples to construct the initial dataset. Subsequently, the synthetic oversampling stage is conducted, which consists of two key steps. In the first step, the Local Outlier Factor (LOF) algorithm is employed for anomaly detection and filtering on the initial data. Based on local density deviations, anomalous samples are identified and removed, yielding a denoised dataset. In the second step, the HCAB-SMOTE adaptive oversampling method is applied to cluster the minority-class fault samples in the imbalanced dataset into groups, and high-quality synthetic samples are generated within each cluster, thereby constructing a class-balanced training dataset. Finally, the Bagging ensemble learning framework is adopted to perform multiple rounds of adaptive sampling training on the balanced dataset for base classifiers. The final diagnostic results are obtained through the fusion of predictions from multiple models, and the effectiveness of the proposed method is verified via multi-dimensional evaluation metrics. The proposed method framework is shown in Figure 2.

**Figure 2:**
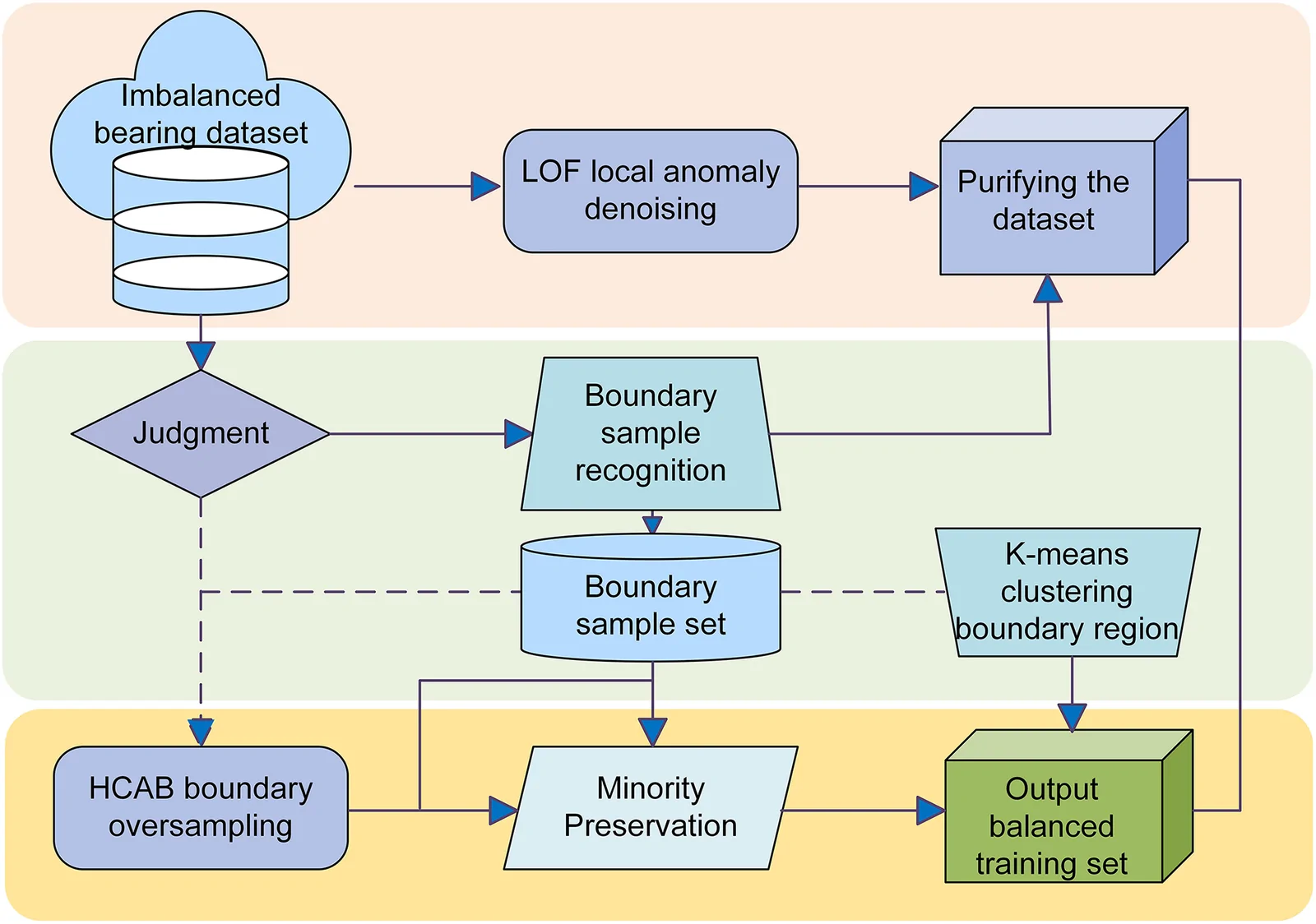
The method framework of HCAB-SMOTE-LOF

This method specifically addresses the challenges of outliers in the original feature space, imbalanced sample distribution, and insufficient model generalization capability in rotating machinery bearing fault diagnosis, thereby effectively enhancing the stability and accuracy of multi-class fault identification.

### Experimental verification

In this section, two case studies are conducted. The first study involves the acquisition of bearing vibration signals from a laboratory test rig, while the second study analyzes vibration signals measured along different directions. Based on these two sets of vibration data, the issues of small sample sizes and imbalanced class distributions are simulated. The effectiveness of the proposed method in addressing these problems through oversampling and subsequent balanced dataset construction is verified via fault identification. In addition, the proposed method is compared with several commonly used approaches in this field, including SMOTE, Borderline-SMOTE, and HCAB-SMOTE.

### Performance analysis of HCAB-SMOTE-LOF on laboratory bearing dataset

In this section, the bearing dataset from the PT500mini fault test rig is employed, and the bearing vibration signals are utilized for validation. Figure 3 illustrates the experimental platform for collecting bearing vibration signals, which consists of a motor, coupling, bearings, and a rotor. The adjustable-speed rotating motor used on the test platform generates broadband mechanical noise due to unbalanced vibration transmitted through the rigidly connected coupling. To simulate single-point faults in bearings, square grooves are machined on the outer race, inner race, and rolling elements using wire electrical discharge machining. The vibration signals collected during the experiments are used to verify the fault diagnosis performance.

**Figure 3:**
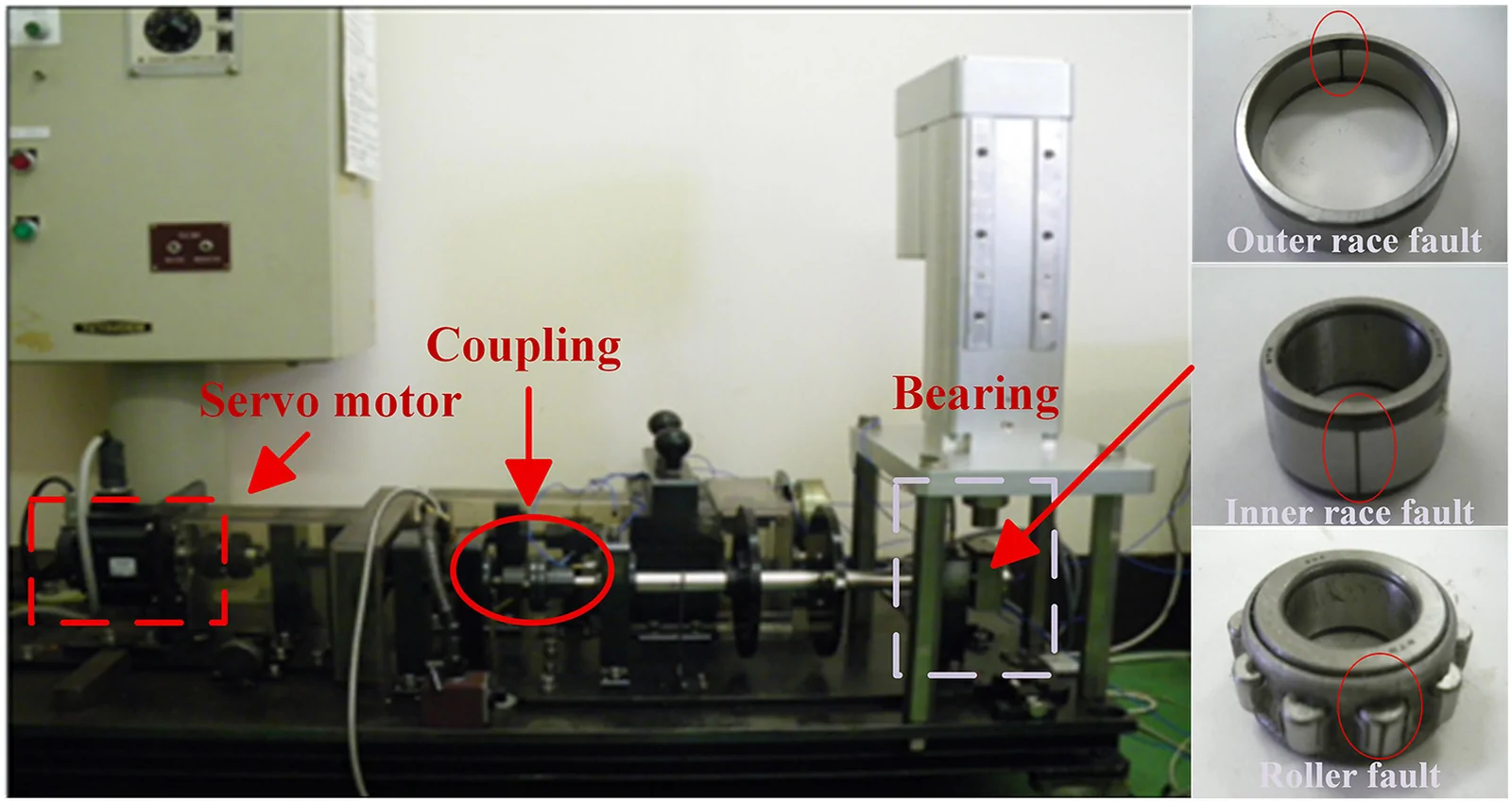
Experimental platform

Acceleration sensors are adopted for vibration signal acquisition. During data collection, the bearing rotational speed is maintained at 1500 r/min, and the sampling frequency is set to 100 kHz. The fault types include roller fault, outer race fault, and inner race fault. For each experiment, the corresponding faulty bearing is installed separately on the test rig. The dataset comprises one normal bearing signal sample and three fault signal samples.

In this experiment, one normal bearing state signal and three different fault state signals are used, where the fault states include bearing outer race fault, bearing inner race fault, and roller fault. This dataset is abbreviated as Case I. The bearing rotational speed is maintained at 1500 r/min, and the sampling frequency is 100 kHz. According to the formula *N* = *f*_*s*_×60/*n*, where *N* denotes the number of sampling points per cycle, *f*_*s*_ is the sampling frequency, and *n* is the bearing rotational speed, the number of sampling points per cycle is calculated as 4000. Although the calculated number of sampling points per rotation cycle is 4000, the actual sample length used in our experiments is set to 4096 points. This choice is motivated by two considerations. First, the Fast Fourier Transform (FFT) algorithm, which serves as the foundation for time-frequency feature extraction, requires the signal length to be a power of two for optimal computational efficiency and spectral resolution. 4096 = 2^12^ is a standard FFT length, whereas 4000 would require zero-padding or truncation, potentially introducing spectral leakage. Second, power-of-two sample lengths are widely adopted in rotating machinery fault diagnosis, with 4096 points being a common standard in the literature. Notably, 4096 points cover approximately 1.024 rotation cycles, fully encompassing the complete periodic signal while maintaining compatibility with FFT-based analysis. The additional 96 points account for only 2.4% of a rotation cycle and do not affect the periodic characteristics of the signal. In addition, the waveforms of the four fault types exhibit distinct differences, and the data characteristics and patterns are presented in Figure 4.

**Figure 4:**
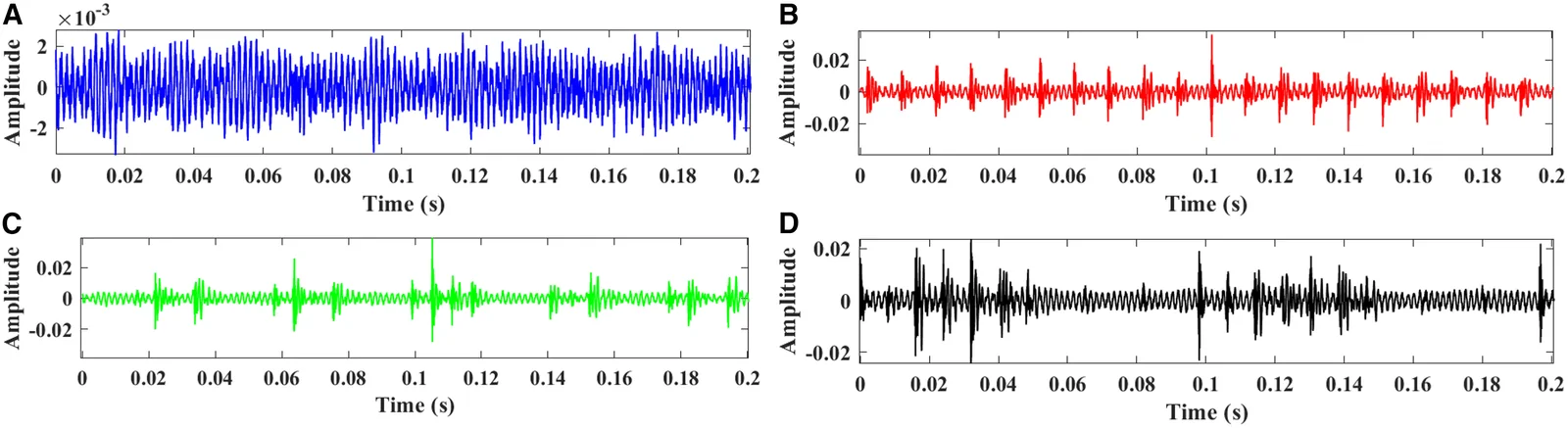
Waveforms of four types of faults (A) Normal. (B) Outer race fault. (C) Inner race fault. (D) Roller fault.

Table 1 summarizes the specific information for each data group. To simulate the imbalanced distribution between normal and fault data in actual production processes, the signals of each state are segmented into samples, with fault training samples set to 20 and each sample length set to 4096 data points. As mentioned in the introduction, the sample size of bearing fault categories is usually smaller than that of normal bearing samples. Therefore, two training datasets were constructed with imbalance ratios of 1:7 and 1:10, respectively, to simulate real operating conditions. According to the two imbalanced ratios, the normal training samples are divided into 140 and 200, and the test samples are all 100.

**Table 1:** The bearing data information of Case I

Fault mode	RPM	Training samples (1:7)	Training samples (1:10)	Test samples
Normal	1500	140	200	100
Inner race fault	1500	20	20	100
Outer race fault	1500	20	20	100
Roller fault	1500	20	20	100

After applying filtering to the data and extracting time-frequency features from each dataset, a data quality assessment is conducted to verify the effectiveness of the LOF-based outlier removal strategy. As described in the method details, the LOF algorithm is applied exclusively to the majority-class data with the parameter set to *α* = 2.5, ensuring that only samples exhibiting significant local density deviations are identified as outliers, thereby avoiding inadvertent deletion of minority-class samples. To visually illustrate the spatial distribution of the detected outliers and the subsequent improvement in data quality, principal component analysis (PCA) is employed to project the original high-dimensional feature space onto the first three principal components. These three components together retain more than 95% of the total variance, ensuring that the low-dimensional visualization reliably reflects the structure of the high-dimensional data. Meanwhile, to quantitatively evaluate the improvement in class separability after LOF-based outlier removal, we compute three metrics: silhouette coefficient, inter-class distance, and intra-class distance. Define the contour coefficient *S* using Equation 1.

(Equation 1) $$S=\frac{1}{n}\sum_{i=1}^{n}\frac{b\left(i\right)-a\left(i\right)}{\max\left\{a\left(i\right),b\left(i\right)\right\}}$$

Where *a*(*i*) is the average distance from sample *i* to other samples in the same class, and *b*(*i*) is the minimum average distance from sample *i* to samples in other classes. A higher *S* indicates better cluster compactness and inter-class separation. The inter-class distance *D*_inter_ and intra-class distance *D*_intra_ are calculated using Equation 2 and Equation 3.

(Equation 2) $$D_{\text{inter}}=\frac{1}{C\left(C-1\right)}\sum_{i=1}^{C}\sum_{\begin{array}{l} j=1\\ j\neq i \end{array}}^{C}∥\mu_{i}-\mu_{j}{∥}_{2}$$

(Equation 3) $$D_{\text{intra}}=\frac{1}{C}\sum_{i=1}^{C}\frac{1}{n_{i}}\sum_{x\in D_{i}}∥x-\mu_{i}{∥}_{2}$$

Where *C* is the number of classes, *μ*_*i*_ is the centroid of class *i*, *n*_*i*_ is the number of samples in class *i*, and *D*_*i*_ is the set of samples belonging to class *i*. Higher *D*_inter_ indicates better class separation, while lower *D*_intra_ indicates tighter intra-class clustering.

Under the imbalance ratio of 1:7, the LOF algorithm detects two outliers among the 140 normal samples, accounting for 1.4% of the majority class. Figures 5A and 5B present the corresponding PCA projection results. Figure 5A shows that the two outliers are marked by red circles, with one outlier located primarily near the boundary region of the minority-class cluster and the other situated in a sparsely populated region of the feature space. As shown in Figure 5B, after LOF-based filtering, the distribution of the normal class becomes more concentrated, and the overlapping area between the normal class and the minority classes is significantly reduced, indicating improved class separability.

**Figure 5:**
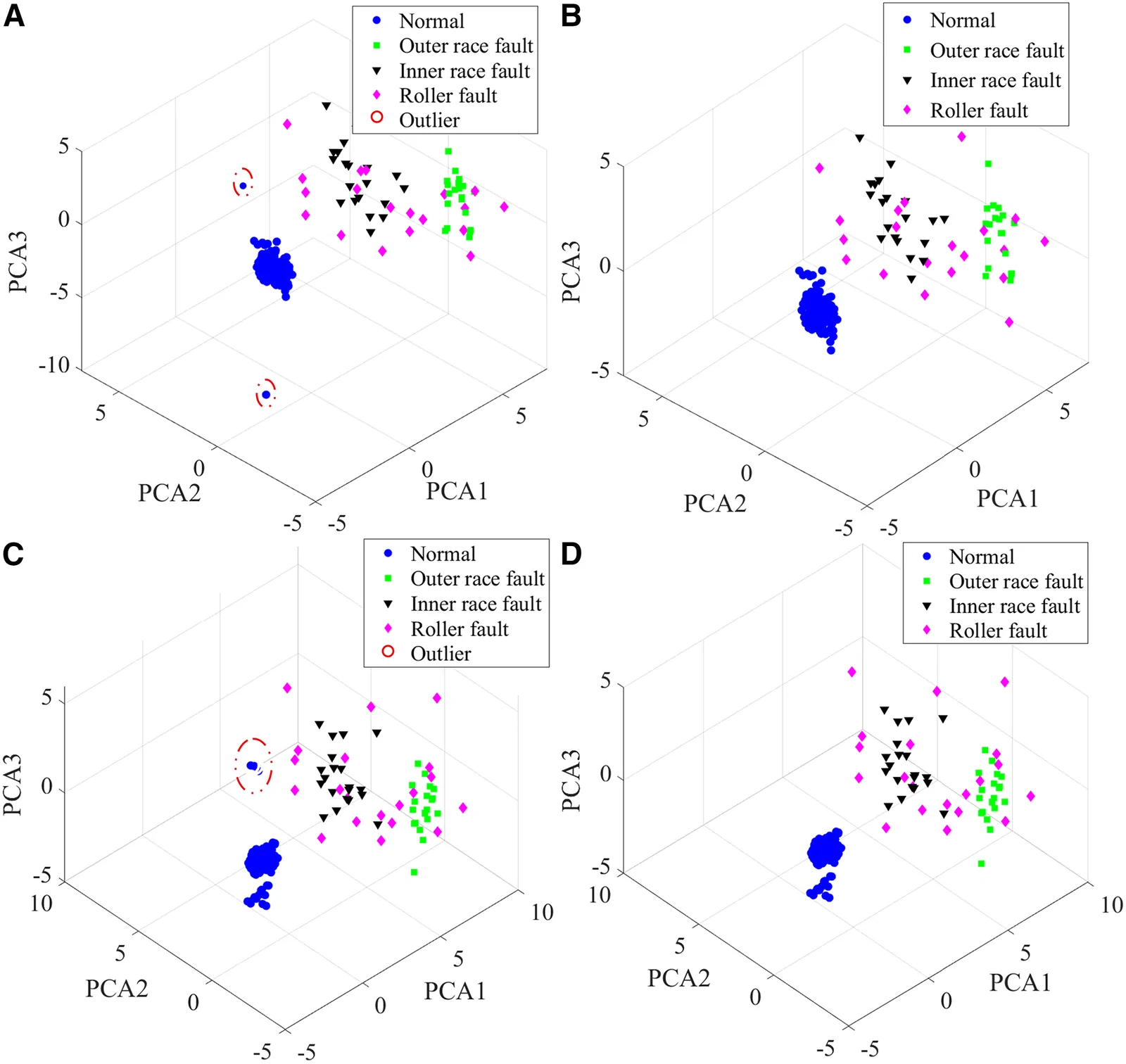
PCA-based 3D visualization of the Case I training data before and after outlier removal (A) Original dataset (imbalance ratio of 1:7). (B) Dataset after outlier removal (1:7). (C) Original dataset (imbalance ratio of 1:10). (D) Dataset after outlier removal (1:10).

Under the imbalance ratio of 1:10, the LOF algorithm identifies three outliers among the 200 normal samples, accounting for 1.5% of the majority class. Similar to the 1:7 case, as shown in Figure 5C, these three outliers are marked by red circles and are clearly located at the periphery of the normal-class cluster. Notably, these three outliers are situated close to the roller fault cluster. Such spatial distribution can adversely affect sample generation, as outliers near the boundary may mislead the synthetic sample generation process, leading to interpolation operations in ambiguous regions and consequently producing synthetic samples that deviate from the true fault distribution. As shown in Figure 5D, the clustering of the normal class becomes more compact, and the three boundary outliers have been eliminated. The class boundaries among the four health conditions also become more distinct, particularly between the normal class and the inner race fault class. This demonstrates that the LOF module can successfully target and remove noise exclusively from the majority class without compromising the integrity of the minority-class fault samples.

According to Table 2, after outlier removal based on LOF, the silhouette coefficient increased from 0.5707 to 0.5792 (+1.48%) and from 0.6145 to 0.6282 (+2.23%) in two imbalanced ratios of 1:7 and 1:10, respectively. Indicating enhanced cluster compactness and inter-class separation. The inter-class distance increases from 5.2920 to 5.3192 (+0.51%) and from 5.7003 to 5.7660 (+1.15%) in two imbalanced ratios of 1:7 and 1:10, respectively. This confirms that class boundaries become more distinct. Meanwhile, the intra-class distance of the normal class decreases from 3.7532 to 3.7189 (−0.91%) and from 4.0389 to 3.9880 (−1.26%) in two imbalanced ratios of 1:7 and 1:10, respectively. This demonstrates that the majority-class samples become more tightly clustered after outlier removal. These quantitative results are consistent with the visual observations in Figure 5, providing strong evidence that the removal of outliers in LOF effectively improves data quality.

**Table 2:** Summarized the changes in these three indicators before and after outlier removal based on LOF in Case I under imbalanced ratios of 1:7 and 1:10

Radio	Metric	Before LOF	After LOF	Improvement
1:7	silhouette coefficient	0.5707	0.5792	+1.48%
1:7	inter-class distance	5.2920	5.3192	+0.51%
1:7	intra-class distance (normal)	3.7532	3.7189	−0.91%
1:10	silhouette coefficient	0.6145	0.6282	+2.23%
1:10	inter-class distance	5.7003	5.7660	+1.15%
1:10	intra-class distance (normal)	4.0389	3.9880	−1.26%

It is worth noting that the number of removed outliers varies with the imbalance ratio, reflecting the differences in local density patterns under different class proportions. In both cases, the proportion of removed samples relative to the training data remains minimal (under 3%), indicating that the LOF module adopts a conservative and targeted cleaning operation. This strategy ensures that the vast majority of valid samples are retained, thereby preventing the loss of valuable fault information, while effectively eliminating boundary noise that could otherwise degrade the performance of subsequent oversampling methods.

Table 3 presents the classification accuracies of the five oversampling strategies under imbalance ratios of 1:7 and 1:10. All experiments are evaluated using a Bagging ensemble classifier comprising 50 base estimators. For the proposed HCAB-SMOTE-LOF method, the results are obtained on the training set after LOF-based cleaning, followed by HCAB-SMOTE oversampling, whereas the results of the other four methods are obtained on the original training sets without any preprocessing. Each method is repeated ten times, and the average accuracy is reported to mitigate the influence of randomness.

**Table 3:** Case I: Comparison of classification accuracy (%) between HCAB-SMOTE-LOF and other oversampling methods using Bagging ensemble

Radio	Original	SMOTE	Borderline-SMOTE	HCAB- SMOTE	HCAB-SMOTE-LOF
1:7	0.745	0.902	0.920	0.945	**0.958**
1:10	0.748	0.907	0.935	0.943	**0.963**

Bold values indicate the best performance among the compared methods.

As shown in Figure 6, the proposed method achieves the highest accuracy under both imbalance ratios of 1:7 and 1:10. Specifically, under the 1:10 ratio, the proposed method improves the accuracy by 2% over HCAB-SMOTE (94.30%) and significantly outperforms Original, SMOTE, and Borderline-SMOTE. These results indicate that the proposed method exhibits more pronounced performance advantages as the degree of class imbalance becomes more severe.

**Figure 6:**
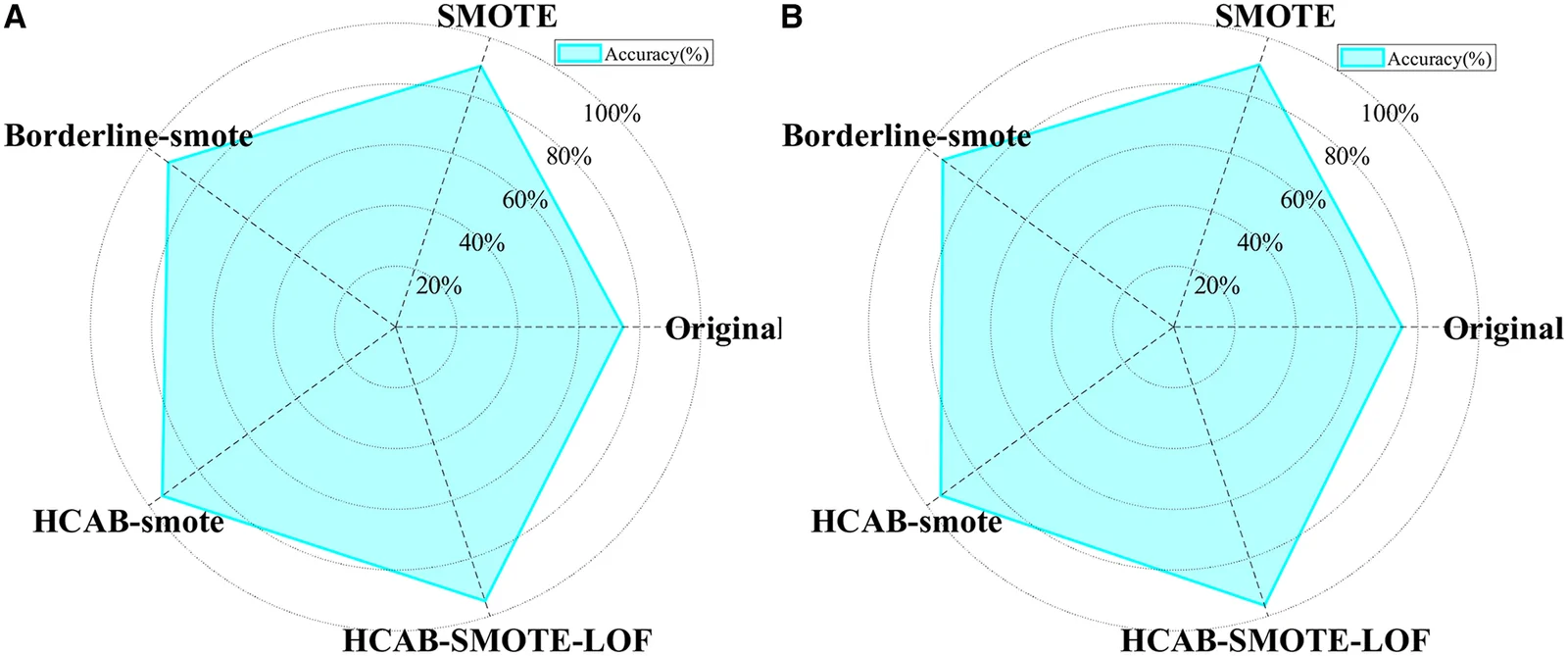
Case I: Accuracy results of fault detection using different methods with Bagging ensemble under two imbalance ratios (A) 1:7. (B) 1:10.

To further evaluate the superiority of the proposed method, additional metrics including precision, recall, F1-score, and G-mean are calculated. As shown in Figures 7A and 7B, which present the results under imbalance ratios of 1:7 and 1:10, respectively, HCAB-SMOTE-LOF consistently outperforms the other methods across all metrics. Under the 1:7 imbalance ratio, the proposed method achieves precision, recall, F1-score, and G-mean of 95.20%, 94.90%, 94.80%, and 97.40%, respectively. It is worth noting that as the imbalance ratio increases, the precision, recall, F1-score, and G-mean of the Original method combined with the Bagging ensemble all increase, suggesting that the model achieves the best fault identification performance when more samples are available.

**Figure 7:**
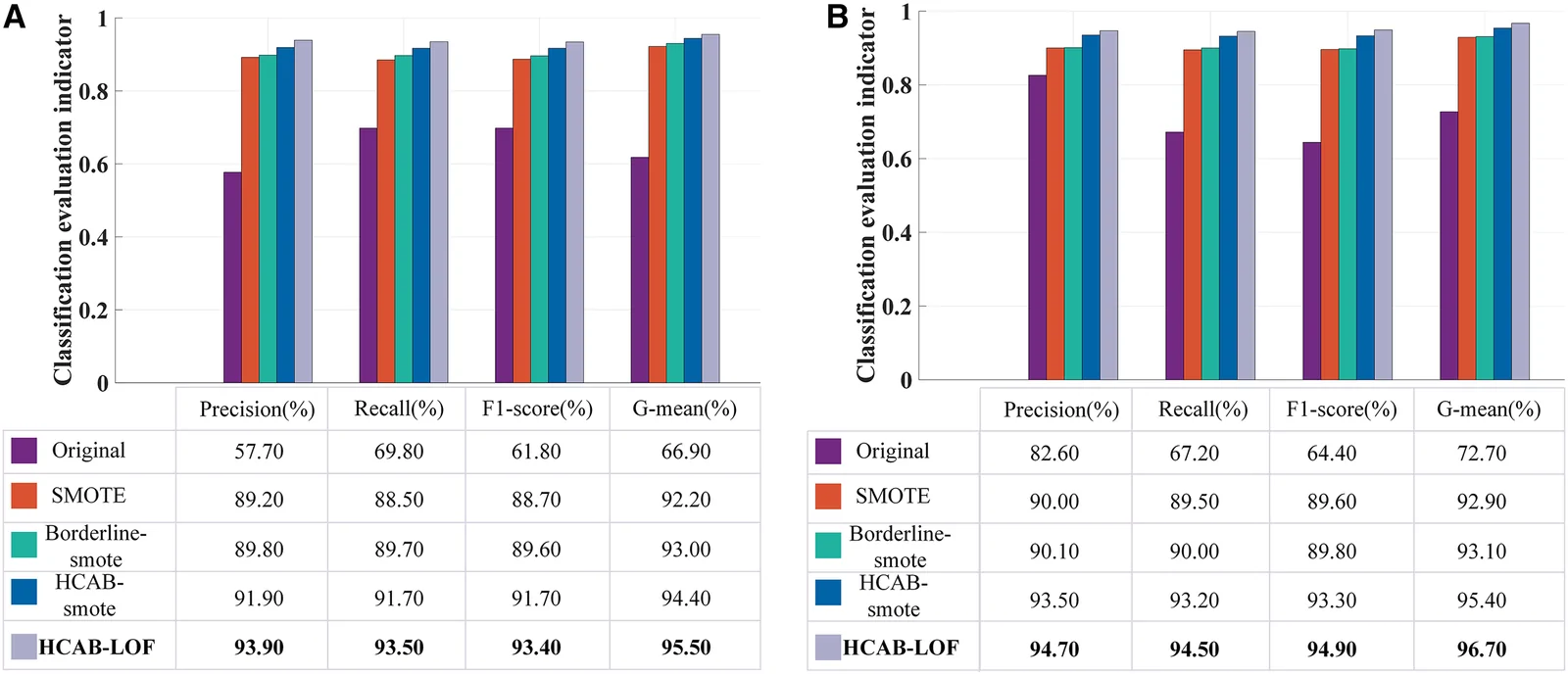
Case I: Quantitative detection results using different methods with Bagging ensemble under two imbalance ratios (A) 1:7. (B) 1:10.

### Performance analysis of HCAB-SMOTE-LOF on the motor vibration and audio dataset of the University of Ottawa

The motor vibration and audio dataset from the University of Ottawa is derived from a modified SpectraQuest Mechanical Fault and Rotor Dynamics Simulator test rig.^21^ Figure 8 illustrates the composition of the experimental setup, which includes an induction motor, a variable frequency drive (VFD), three single-axis accelerometers, and a microphone. The data acquisition system employs a National Instruments USB-6212 data acquisition module to establish the connection between the sensors and the computer. The accelerometers are responsible for collecting vibration and temperature signals at the drive end and the shaft system, while the microphone is used to record acoustic data. The rotational speed of the motor is measured by the variable frequency drive. The data acquisition process is controlled by LabVIEW software, with each acquisition lasting 10 s. All signals are sampled at a rate of 42 kHz.^22^

**Figure 8:**
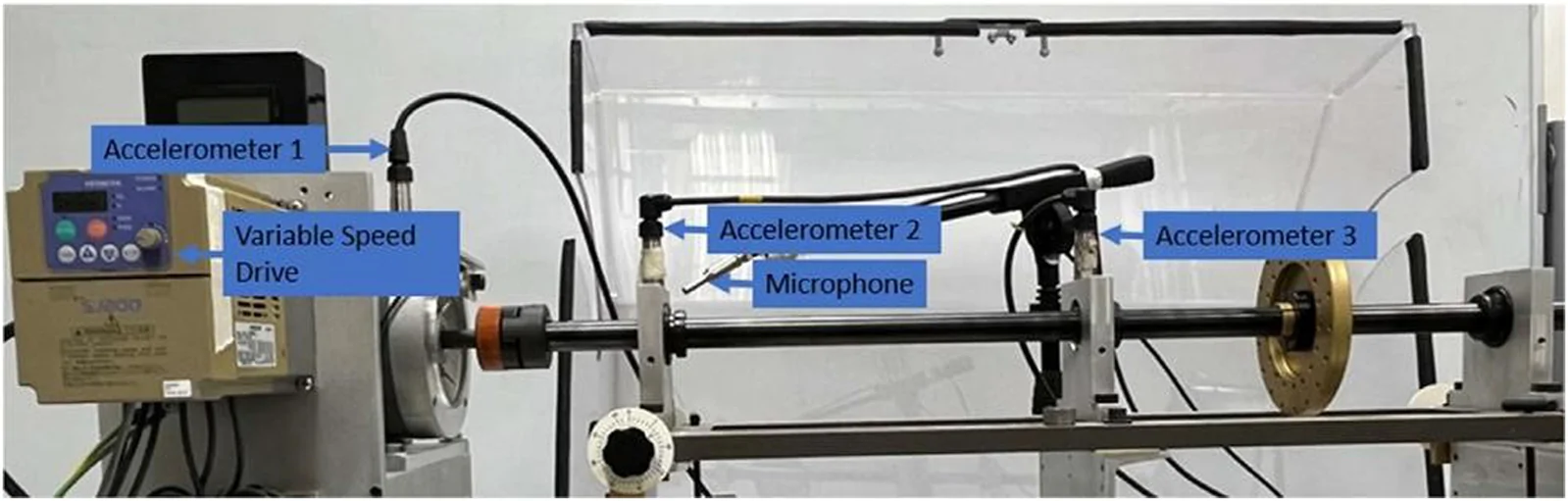
Case II experimental setup

Each data file consists of multiple columns of time-series measurements. The first column contains data from the accelerometer at the motor drive end (PCB, model 603C01), the second column records acoustic data, the third column contains measurements from the second accelerometer (PCB, model 623C01) mounted on the shaft bearing housing near the drive end, the fourth column contains data from the third accelerometer (PCB, model 623C01) located at the farthest position from the drive end on the shaft bearing housing, and the fifth column records temperature data from the motor surface at the drive end. The dataset comprises samples from a healthy induction motor as well as various fault states, which can be categorized into the following two types.

(1) Electrical faults: including stator winding faults (SW), voltage unbalance (VU), and broken rotor bars (KA).
(2) Mechanical faults: including rotor unbalance (RU), rotor misalignment (RM), bowed rotor (BR), and fault bearings (FB).

Thus, the dataset contains a total of eight classes, corresponding to seven fault states and one normal motor state. The induction motor can operate under both constant-frequency and variable-frequency conditions. The constant frequencies range approximately from 15 Hz, 30 Hz, 45 Hz, and 60 Hz, while the variable frequency ranges include intervals such as 15–45 Hz, 30–60 Hz, 45–15 Hz, and 60–30 Hz. This study exclusively utilizes the constant-speed operation portion of the Ottawa dataset, and all subsequent discussions are based on this subset. The motor can operate under no-load or loaded conditions, with the latter achieved by symmetrically securing ten bolts to a disk mounted on the motor shaft. The dataset file names follow the format “letter-letter-number-number”, where the first two letters indicate the motor condition (e.g., “H” for healthy, “R” for rotor fault, and so forth), the first number denotes the motor speed setting (e.g., 1 = 15 Hz, 4 = 60 Hz), and the second number indicates the load condition (“0” for no load, “1” for load). For example, “S-W-4-1” denotes a loaded stator winding fault under 60 Hz operating conditions. The structure of the constant-speed dataset under both no-load and loaded conditions is detailed in Table 4.

**Table 4:** Dataset annotation under constant speed conditions under different fault modes

Motor load condition	Fault mode	Constant speed			
		15 Hz	15 Hz	45 Hz	60 Hz
Unloaded	healthy (H)	H-H-1-0	H-H-2-0	H-H-3-0	H-H-4-0
Unloaded	rotor unbalance (RU)	R-U-1-0	R-U-2-0	R-U-3-0	R-U-4-0
Unloaded	rotor misalignment (RM)	R-M-1-0	R-M-2-0	R-M-3-0	R-M-4-0
Unloaded	stator winding fault (SW)	S-W-1-0	S-W-2-0	S-W-3-0	S-W-4-0
Unloaded	voltage unbalance (VU)	V-U-1-0	V-U-2-0	V-U-3-0	V-U-4-0
Unloaded	bowed rotor (BR)	B-R-1-0	B-R-2-0	B-R-3-0	B-R-4-0
Unloaded	broken rotor bars (KA)	K-A-1-0	K-A-2-0	K-A-3-0	K-A-4-0
Unloaded	faulty bearings (FB)	F-B-1-0	F-B-2-0	F-B-3-0	F-B-4-0
Loaded	healthy (H)	H-H-1-1	H-H-2-1	H-H-3-1	H-H-4-1
Loaded	rotor unbalance (RU)	R-U-1-1	R-U-2-1	R-U-3-1	R-U-4-1
Loaded	rotor misalignment (RM)	R-M-1-1	R-M-2-1	R-M-3-1	R-M-4-1
Loaded	stator winding fault (SW)	S-W-1-1	S-W-2-1	S-W-3-1	S-W-4-1
Loaded	voltage unbalance (VU)	V-U-1-1	V-U-2-1	V-U-3-1	V-U-4-1
Loaded	bowed rotor (BR)	B-R-1-1	B-R-2-1	B-R-3-1	B-R-4-1
Loaded	broken rotor bars (KA)	K-A-1-1	K-A-2-1	K-A-3-1	K-A-4-1
Loaded	faulty bearings (FB)	F-B-1-1	F-B-2-1	F-B-3-1	F-B-4-1

This dataset is abbreviated as Case II. This case study covers four fault types, namely H (Healthy), FB (Faulty Bearings), RM (Rotor Misalignment), and BR (Bowed Rotor), under a constant frequency of 15 Hz and no-load operating conditions. Figure 9 presents the normalized vibration waveforms acquired from the first accelerometer at the operating frequency of 15 Hz for the four types of data. The detailed composition of the dataset in Case II is provided in Table 5. The parameter fault training samples is set to 20, with each sample containing 1024 data points. Other sampling parameters, including the imbalance ratio and test samples, remain consistent with those in Case I.

**Figure 9:**
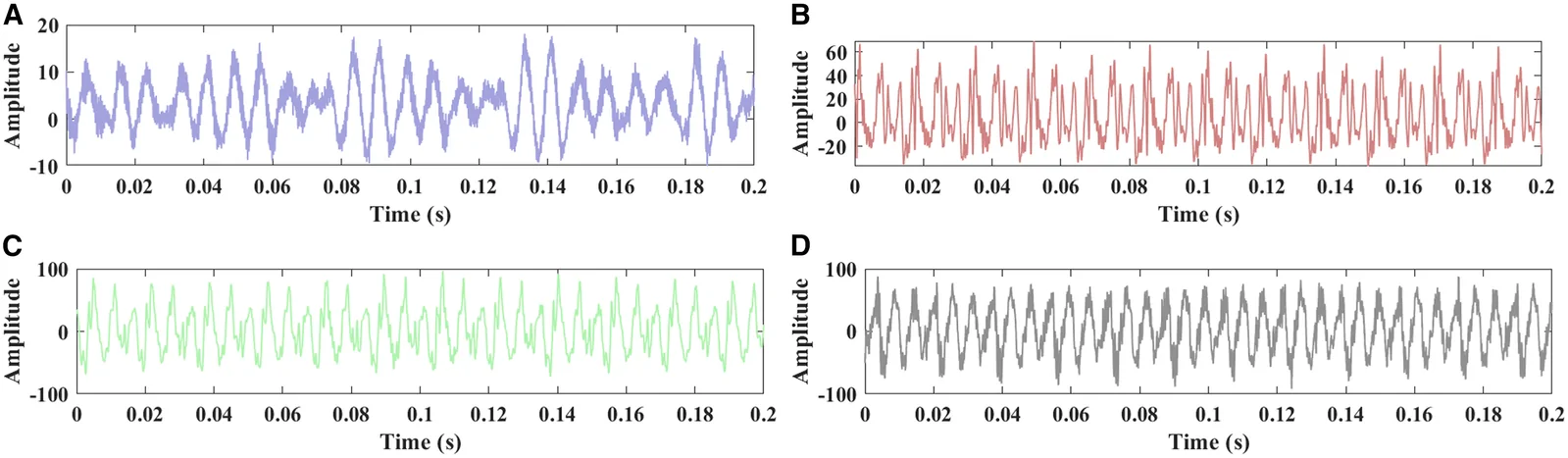
Waveforms of four types of faults (A) Healthy condition. (B) Bowed rotor (BR). (C) Rotor misalignment (RM). (D) Faulty bearings (FB).

**Table 5:** Case II: Sample information for each input channel in the constant-speed dataset

Fault mode	Motor load condition	Training samples (1:7)	Training samples (1:10)	Test samples
Healthy (H)	Unloaded	140	200	100
Bowed Rotor (BR)	Unloaded	20	20	100
Rotor Misalignment (RM)	Unloaded	20	20	100
Faulty Bearings (FB)	Unloaded	20	20	100

Following the preprocessing protocol established in Case I, the LOF algorithm is applied exclusively to the majority-class samples with the same parameter configuration. In order to quantitatively analyze the improvement of class separability after removing outliers based on LOF, three indicator values are calculated: silhouette coefficient, inter-class distance, and intra-class distance. The University of Ottawa vibration dataset comprises four health conditions: healthy, bowed rotor (BR), rotor misalignment (RM), and fault bearings (FB). The LOF algorithm identifies four outliers among the 140 normal training samples under the 1:7 imbalance ratio, and five outliers among the 200 normal samples under the 1:10 imbalance ratio, accounting for 2.9% and 2.5% of the majority class, respectively, and the proportion of removed samples in the total training data is all less than 3%. The detected outliers are primarily located at the boundaries between the normal class and the minority fault clusters. Such boundary-proximity distribution is particularly detrimental to oversampling methods. As the imbalance ratio increases, the class separability in the original data becomes less pronounced, and the increased number of normal samples leads to greater coverage density in the normal class region. Consequently, the impact of outliers on classification boundaries becomes more significant.

To visualize the spatial distribution of these outliers, principal component analysis is employed to project the feature space onto the first three principal components. As shown in Figures 10A and 10C, these outliers are predominantly situated at the boundaries between the normal class and the minority fault classes, particularly in regions close to the BR and RM classes. As illustrated in Figures 10B and 10D, after the LOF-based removal operation, the clustering structure of the normal-class samples is improved, and the distinction between the normal class and the abnormal classes becomes more pronounced. The minority-class samples remain entirely unchanged, indicating that the LOF module processes only majority-class noise while preserving the rare anomalous sample information. Consistent with the findings in Case I, the proportion of removed outliers relative to the training data remains very small, demonstrating that the LOF-based preprocessing operation is both conservative and effective.

**Figure 10:**
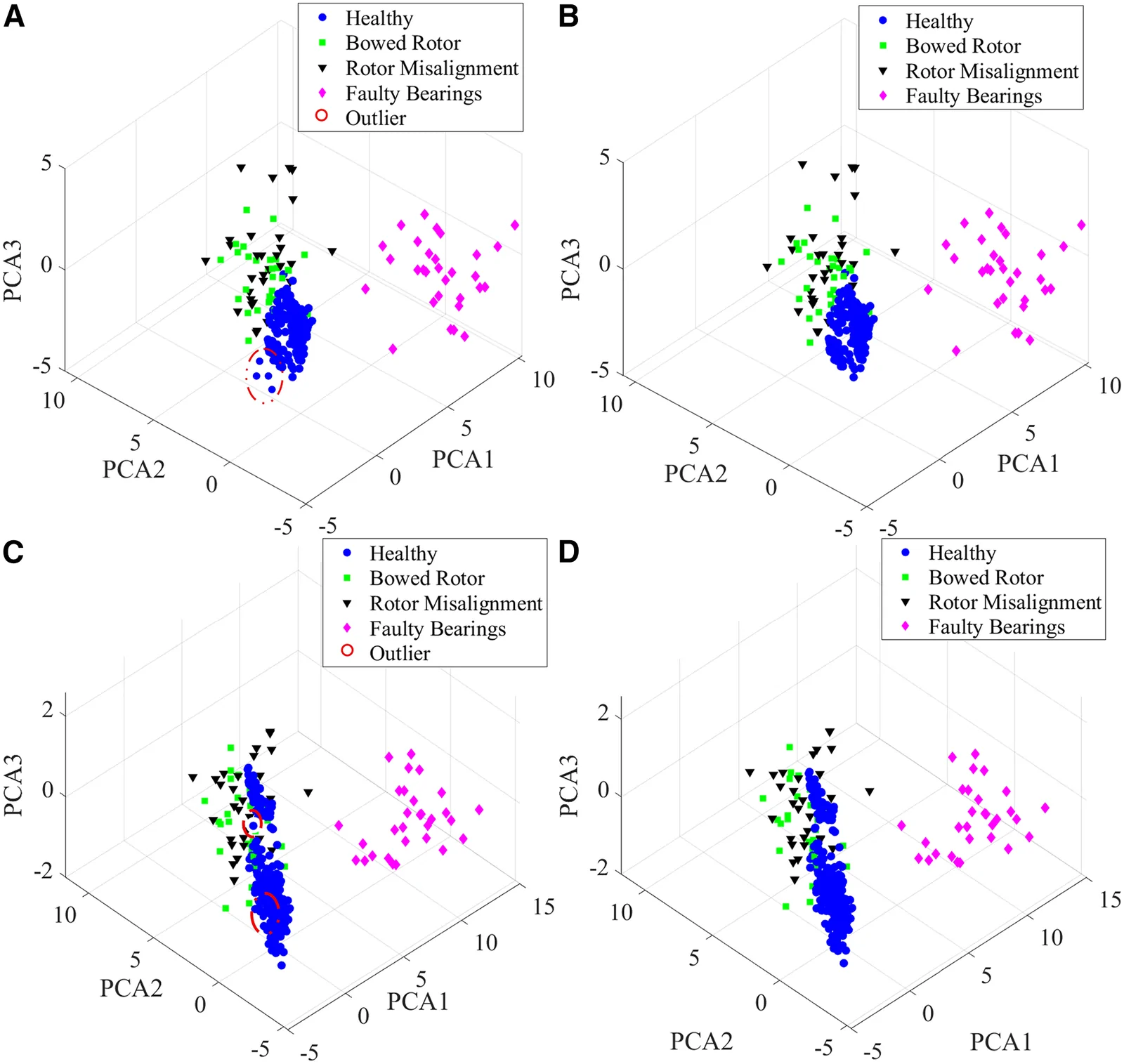
PCA-based 3D visualization of the case II training data before and after outlier removal (A) Original dataset (imbalance ratio of 1:7). (B) Dataset after outlier removal (1:7). (C) Original dataset (imbalance ratio of 1:10). (D) Dataset after outlier removal (1:10).

According to Table 6, for the University of Ottawa dataset, the silhouette coefficient increased from 0.3702 to 0.3717 (+0.40%) and from 0.4089 to 0.4131 (+1.03%) in two imbalanced ratios of 1:7 and 1:10, respectively. The inter-class distance increases from 6.0264 to 6.0362 (+0.16%) and from 6.6612 to 6.7122 (+0.77%) in two imbalanced ratios of 1:7 and 1:10, respectively. Meanwhile, the intra-class distance of the normal class decreases from 4.4952 to 4.4863 (−0.20%) and from 4.8807 to 4.8397 (−0.84%) in two imbalanced ratios of 1:7 and 1:10, respectively. These results further demonstrate the effectiveness of LOF-based preprocessing techniques in improving category separability in different experimental environments.

**Table 6:** Summarized the changes in these three indicators before and after outlier removal based on LOF in Case II under imbalanced ratios of 1:7 and 1:10

Radio	Metric	Before LOF	After LOF	Improvement
1:7	silhouette coefficient	0.3702	0.3717	+0.40%
1:7	inter-class distance	6.0264	6.0362	+0.16%
1:7	intra-class distance (normal)	4.4952	4.4863	−0.20%
1:10	silhouette coefficient	0.4089	0.4131	+1.03%
1:10	inter-class distance	6.6612	6.7122	+0.77%
1:10	intra-class distance (normal)	4.8807	4.8397	−0.84%

As in Case I, LOF cleaning is only used for the proposed HCAB-SMOTE-LOF method, while other strategies are evaluated on the original training set. The other oversampling strategies, including the use of original data, SMOTE, Borderline-SMOTE, and HCAB-SMOTE, are evaluated on the original unprocessed training sets to ensure fair comparison. Only the proposed method benefits from the LOF-cleaned data foundation prior to HCAB-SMOTE oversampling. The comparative evaluation results of the five oversampling strategies are presented in the following subsection.

In this specific experiment, the label prediction results of each oversampling method are presented in Figure 11. Table 7 details the experimental accuracies of the various oversampling methods under two data imbalance ratios (1:7 and 1:10), with all values representing the averages over ten consecutive repeated experiments. Taking the 1:10 imbalance ratio as an example, HCAB-SMOTE-LOF achieves an accuracy of 94.3%, which significantly outperforms the other methods, while the lowest and highest accuracies of Original and HCAB-SMOTE are 67.2% and 93.2%, respectively. Similarly, under the 1:7 imbalance ratio, the proposed method also attains the highest accuracy, leading to conclusions consistent with those drawn in Case I; therefore, further elaboration is omitted.

**Figure 11:**
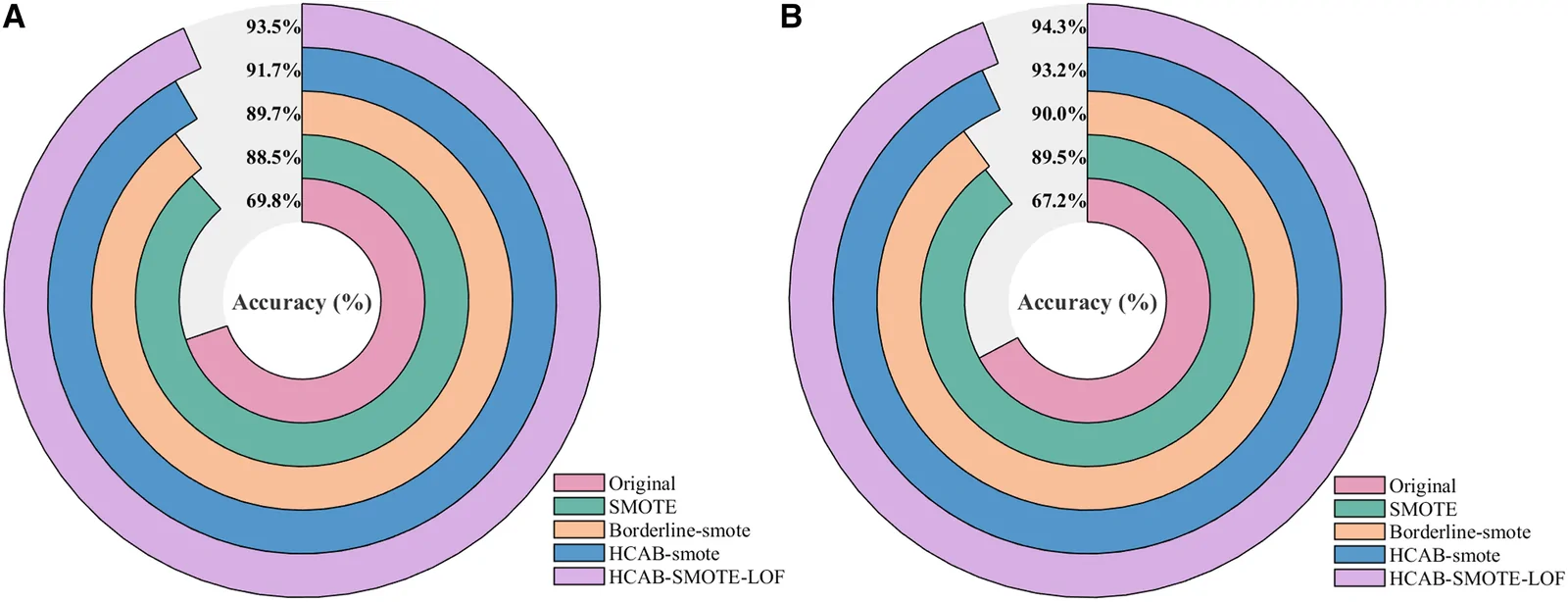
Case II: Accuracy results of fault detection using different methods with Bagging ensemble under two imbalance ratios (A) 1:7. (B) 1:10.

**Table 7:** Case II: Comparison of classification accuracy (%) between HCAB-SMOTE-LOF and other oversampling methods using Bagging ensemble

Radio	Original	SMOTE	Borderline-SMOTE	HCAB- SMOTE	HCAB-SMOTE-LOF
1:7	0.698	0.885	0.897	0.917	**0.935**
1:10	0.672	0.895	0.900	0.932	**0.943**

Bold values indicate the best performance among the compared methods.

Figure 12 presents the precision, recall, F1-score, and G-mean of the four oversampling methods along with HCAB-SMOTE-LOF. As shown in Figure 12A, under the 1:7 imbalance ratio, HCAB-SMOTE-LOF achieves the highest precision, recall, F1-score, and G-mean (93.90%, 93.50%, 93.40%, and 95.50%, respectively), outperforming the other methods. Figure 12B presents the experimental results under the 1:10 imbalance ratio, where HCAB-SMOTE-LOF again achieves the best performance across all metrics. These results demonstrate that, compared with the other methods, HCAB-SMOTE-LOF generates synthetic samples of the highest quality and effectively improves the classification accuracy of the classifier.

**Figure 12:**
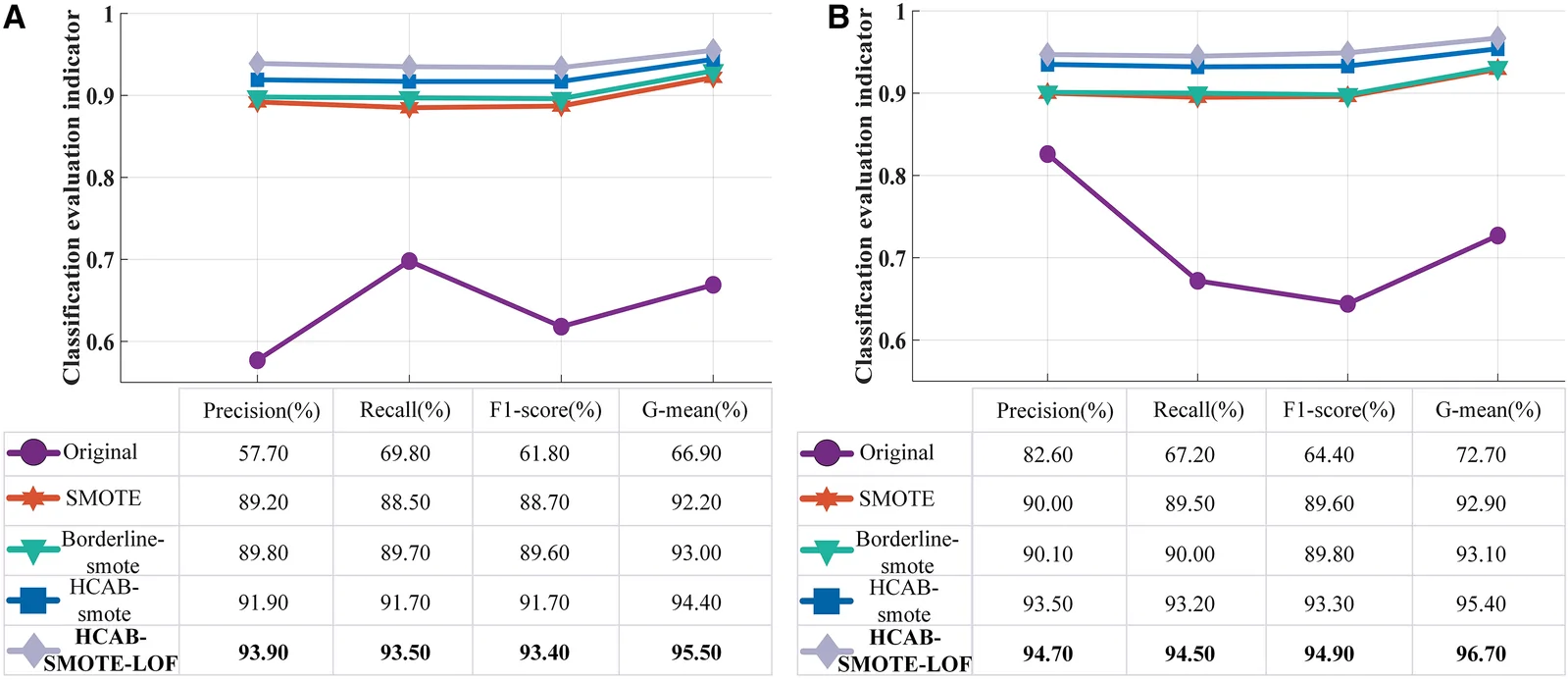
Case II: Quantitative detection results using different methods with Bagging ensemble under two imbalance ratios (A) 1:7. (B) 1:10.

Overall, the two case studies above investigate the classification performance of different oversampling methods combined with a Bagging ensemble. The analysis and comparison of the results demonstrate that the proposed HCAB-SMOTE-LOF method exhibits superior performance in addressing the fault diagnosis problem of bearing data characterized by small sample sizes and class imbalance.

### Ablation experiment

This section systematically analyzes the influence of several key parameters in the proposed HCAB-SMOTE-LOF method on fault diagnosis performance, including the LOF outlier factor threshold *α* and the oversampling ratio *β*. At the same time, modular ablation experiments were conducted on the LOF-based outlier removal module and the HCAB-SMOTE oversampling module. All experiments are conducted using the two imbalanced datasets from Case I, with the Bagging ensemble consistently adopted as the classifier and accuracy serving as the primary evaluation metric. Each experiment is independently run ten times, and the average results are reported to mitigate the influence of randomness.

### Threshold *α* and oversampling ratio *β*

The threshold *α* is used to determine whether a majority-class sample is an outlier: majority-class samples with LOF_*k*_(*x*_*i*_)>*α* are removed. A value of *α* that is too small may lead to the accidental removal of a large number of boundary samples, thereby disrupting the original data distribution, whereas an excessively large *α* fails to effectively eliminate noise. It should also be noted that the LOF-based cleaning operation is constrained by a 3% deletion cap. This cap ensures that the method remains robust even when the dataset contains an unusually high number of boundary samples, as only the most extreme outliers are removed. The oversampling ratio *β* controls the number of new samples generated within each *k*-means cluster, i.e., the number generated = ⌈*β*·*n*_*t*_⌉. If *β* is too small, the class distribution cannot be sufficiently balanced; if *β* is too large, redundant samples may be introduced, potentially leading to overfitting. Figures 13A and 13B illustrate the accuracy trends as *α* varies from 1.5 to 3.5 and *β* varies from 0.6 to 1.5. When *α* = 2.5 and *β* = 1.2, the accuracy reaches its peak under both imbalance ratios of 1:7 and 1:10, achieving 94.8% and 96.3%, respectively. Overall, the proposed method achieves optimal performance when *α*,*β*∈[2.5,1.2], indicating that this parameter interval effectively identifies and removes majority-class noise while preserving sufficient boundary information.

**Figure 13:**
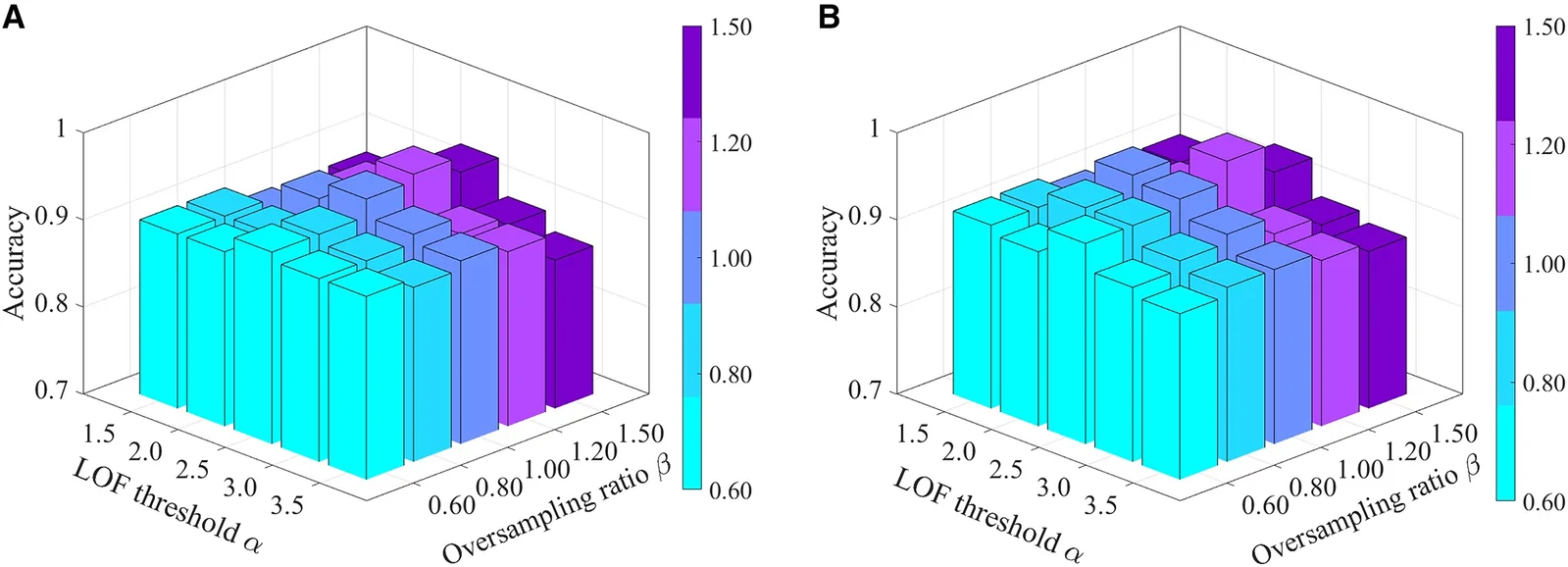
Effects of parameters *α* and *β* on classification accuracy under imbalanced data (A) 1:7. (B) 1:10.

### Modular decomposition ablation experiment

In order to further analyze the independent contributions of the LOF-based outlier removal module and the HCAB-SMOTE oversampling module, we conducted modular decomposition analysis and studied the following eight configurations: (1) Original: No preprocessing, directly classified using Bagging ensemble; (2) Original-LOF: Only LOF-based outlier removal is applied, with no oversampling performed; (3) SMOTE: Standard SMOTE oversampling without LOF preprocessing; (4) SMOTE-LOF: LOF preprocessing followed by SMOTE oversampling; (5) Borderline-SMOTE: Borderline-SMOTE oversampling without LOF preprocessing; (6) Borderline-SMOTE-LOF: LOF preprocessing followed by Borderline-SMOTE oversampling; (7) HCAB-SMOTE: HCAB-SMOTE oversampling without LOF preprocessing; and (8) HCAB-SMOTE-LOF (Proposed Method): Our complete method integrating both LOF and HCAB-SMOTE. This modular design allows us to isolate and evaluate the individual contributions of each component.

According to Table 8, it can be seen that the LOF-only configuration achieves 0.5% and 2.2% accuracy improvements over the original data in Case I respectively in the two imbalanced ratios of 1:7 and 1:10, confirming that LOF-based outlier removal alone enhances classification performance. When LOF is combined with SMOTE, Borderline-SMOTE, and HCAB-SMOTE, it consistently provides additional performance gains, demonstrating the general applicability of LOF as a universal data purification preprocessor. Notably, if the proposed method were a simple combination, SMOTE-LOF and Borderline-SMOTE-LOF would achieve comparable performance. However, the results show that the HCAB-SMOTE-LOF configuration achieves the highest accuracy, indicating the synergistic effect between outlier removal and boundary clustering oversampling rather than an additive one.

**Table 8:** Case I: Comparison of classification accuracy (%) based on LOF module on Bagging ensemble

Method	1:7	1:10
Original	0.745	0.748
Original-LOF	0.750	0.770
SMOTE	0.902	0.907
SMOTE-LOF	0.912	0.922
Borderline-SMOTE	0.920	0.935
Borderline-SMOTE-LOF	0.931	0.945
HCAB-SMOTE	0.945	0.943
HCAB-SMOTE-LOF	**0.958**	**0.963**

Bold values indicate the best performance among the compared methods.

## Discussion

This paper has proposed an intelligent diagnostic method for imbalanced data, termed HCAB-SMOTE-LOF, which integrates outlier-removal-based adaptive oversampling with Bagging ensemble learning to address the challenges in rotating machinery fault diagnosis arising from anomalous outliers in the original feature space, imbalanced class distributions, and insufficient recognition accuracy for minority fault types. In the proposed method, high-pass filtering is first applied to remove low-frequency components from the raw vibration signals, and relevant time-frequency features are subsequently extracted. The local outlier factor algorithm is then employed to eliminate noise and outliers from the original data, thereby ensuring data quality. Following this, HCAB-SMOTE adaptive oversampling is utilized to generate high-quality minority-class fault samples, alleviating the class imbalance problem. Finally, the Bagging ensemble learning framework is adopted to enhance the model’s generalization capability and diagnostic stability. The contribution of this work is not in inventing LOF or HCAB-SMOTE algorithms, but in proposing a design method for imbalanced learning problems under outlier contamination. Meanwhile, through two experimental case studies, the proposed method demonstrates its effectiveness in improving diagnostic accuracy for imbalanced bearing fault data compared with several state-of-the-art oversampling methods. In particular, it exhibits significant advantages in identifying minority fault classes, thereby providing an effective solution to the fault diagnosis problem of rotating machinery bearings under complex operating conditions characterized by small sample sizes and uneven class distributions.

### Limitations of the study

This study was validated on two laboratory datasets—the PT500mini bearing dataset and the University of Ottawa motor dataset. These datasets cover multiple types of faults and two imbalanced ratios (1:7 and 1:10), but may not fully reflect the more complex and variable situations in actual industrial environments. Its applicability and robustness in a wider range of real-world operating environments have not been fully evaluated. In addition, potential factors such as sensor noise, environmental changes, and differences in motor configuration were not considered. Therefore, it is recommended that future research use large-scale real fault datasets to validate the proposed method and explore its extension to lightweight and universal diagnostic frameworks for practical applications in rotating machinery.

## Resource availability

### Lead contact

Requests for further information and resources should be directed to and will be fulfilled by the lead contact, Xuewei Song (tianya0218s@126.com).

### Materials availability

This study did not generate new unique reagents.

### Data and code availability

• The University of Ottawa electric motor vibration dataset analyzed in this study is publicly available at its official repository (https://doi.org/10.1016/j.dib.2024.110144).
• The laboratory bearing vibration data collected on the PT500mini test rig in this study are available from the lead contact upon request.
• All original code has been deposited on GitHub and is publicly available at https://github.com/yanzhijia-s/Fault-Diagnosis.git as of the date of publication.
• Any additional information required to reanalyze the data reported in this paper is available from the lead contact upon request.

## Acknowledgments

This work was supported in part by the 10.13039/501100001809National Natural Science Foundation of China under grant 52401418 and grant 52201355; in part by the Guangdong Province Overseas Famous Teacher Project under grant MS202500036.

## Author contributions

Conceptualization, Z.Q.L.; methodology, Z.Q.L. and Z.J.Y.; investigation, Z.Q.L., Z.J.Y., B.Z.J., and X.W.S.; writing – original draft, Z.Q.L. and Z.J.Y.; writing – review and editing, Z.J.Y. and X.W.S.; funding acquisition, Z.Q.L. and X.W.S.; resources, B.Z.J.; supervision, Z.Q.L., B.Z.J., and X.W.S.

## Declaration of interests

The authors declare no competing interests.

## STAR★Methods

### Key resources table

**Table:** 

REAGENT or RESOURCE	SOURCE	IDENTIFIER
**Software and algorithms**		

MATLAB R2023	MathWorks	https://www.mathworks.com/products/matlab.html

**Other**		

University of Ottawa Electric Motor Dataset	This paper	https://data.mendeley.com/datasets/msxs4vj48g/1

### Experimental model and study participant details

This study does not involve biological experimental models or human participants. Collect normal state data and fault state data from actual rotating machinery equipment. Therefore, biological attributes (species, genotype, age, gender) and ethical certification requirements are not applicable.

### Method details

#### SMOTE

In classification tasks, the problem of data imbalance tends to bias the model toward predicting the majority class, thereby compromising its overall performance. To address this issue, researchers have introduced oversampling techniques. These methods aim to balance the class distribution by augmenting the number of minority-class samples, so that the class sizes become more comparable, and the model is less prone to favor the majority class. Oversampling approaches include simple replication of minority instances, as well as the generation of new synthetic minority samples through interpolation and other related strategies.

The Synthetic Minority Over-sampling Technique (SMOTE) generates new samples by performing linear interpolation on the features of minority-class instances and randomly creating synthetic instances between neighboring minority points. Formally, let the minority-class sample set be denoted as *S* with a total of *n* samples. For each minority sample *x*_*i*_∈*S*(*i*=1,2,…,*n*), its *k* nearest neighbors are first determined based on a chosen distance metric. Let $x_{i}^{N}$ represent the *N*-th nearest neighbor of *x*_*i*_, where *N*=1,2,…,*k*. Then, for each *x*_*i*_, one of its nearest neighbors *x*_*j*_ is randomly selected, and a synthetic sample *x*_*new*_ is generated according to Equation 4.

(Equation 4) $$x_{new}=x_{j}+\lambda \cdot \left(x_{neighbor}-x_{j}\right)$$

Where *λ* is a uniform random number in the interval [0,1]. Finally, to obtain the desired number of minority samples, the above procedure is repeated multiple times in accordance with the required oversampling ratio, thereby effectively augmenting the dataset.

#### HCAB-SMOTE-LOF

Although traditional oversampling methods have improved the quality of generated samples through clustering and boundary detection, they still fail to effectively identify and remove noisy samples present in the majority class of the original training set. Such noisy samples can mislead the synthesis process, causing the newly generated samples to deviate from the true fault distribution. To address this issue, this paper proposes a method that combines the Hybrid Clustered Affinitive Borderline Synthetic Minority Over-sampling Technique with Local Outlier Factor (HCAB-SMOTE-LOF), which introduces the Local Outlier Factor (LOF) prior to sample generation via HCAB-SMOTE to purify the training set. This design ensures that LOF does not merely remove outliers, but redefines the reliable boundary region for HCAB-SMOTE. Similarly, HCAB-SMOTE does not oversample blindly, but generates samples within purified and cluster-consistent minority-class regions, further enhancing the reliability and distributional rationality of the synthesized samples. The proposed HCAB-SMOTE-LOF framework consists of four sequential modules, as illustrated in Figure S2: LOF-based majority cleaning, Boundary sample identification, Minority preservation, and HCAB-SMOTE oversampling. The following subsections provide detailed mathematical formulations for each module.

Specifically, let the original training set be denoted as $D={\left\{\left(x_{i},y_{i}\right)\right\}}_{i=1}^{N}$, where *x*_*i*_ϵ*R*^*d*^ is the feature vector, *N* is the total number of samples, and *y*_*i*_∈{0,1,…,*C*-1} is the corresponding class label. Let *M* and *S* represent the sets of majority-class and minority-class samples, respectively. For each sample *x*_*i*_, we define *d*_*k*_(*x*_*i*_) as the Euclidean distance from *x*_*i*_ to its *k*-th nearest neighbor. For any two samples *x*_*i*_ and *x*_*j*_, their reachability distance is defined as given in Equation 5.

(Equation 5) $$\text{reach}-{\text{dist}}_{k}\left(x_{i},x_{j}\right)=\max\left(d_{k}\left(x_{j}\right),∥x_{i}-x_{j}∥\right)$$

Where ∥*x*_*i*_-*x*_*j*_∥ denotes the Euclidean distance between *x*_*i*_ and *x*_*j*_. For sample *x*_*i*_, its local reachability density is defined as the reciprocal of the average reachability distance over its *k* nearest neighbors *N*_*k*_(*x*_*i*_), as shown in Equation 6.

(Equation 6) $$lrd_{k}\left(x_{i}\right)=\frac{1}{\frac{1}{k}\sum_{x_{j}\in N_{k}\left(x_{i}\right)}\text{reach}-{\text{dist}}_{k}\left(x_{i},x_{j}\right)}$$

Where *N*_*k*_(*x*_*i*_) denotes the *k* nearest neighbors of *x*_*i*_. The local outlier factor of sample *x*_*i*_ is then defined as the average ratio of the local reachability densities of its neighbors to its own local reachability density, as given in Equation 7.

(Equation 7) $${\text{LOF}}_{k}\left(x_{i}\right)=\frac{1}{k}\sum_{x_{j}\in N_{k}\left(x_{i}\right)}\frac{{\text{lrd}}_{k}\left(x_{j}\right)}{{\text{lrd}}_{k}\left(x_{i}\right)}$$

A higher LOF value indicates that the sample has a lower density relative to its neighborhood, implying a greater likelihood of being an outlier. A threshold *a*>0 is set. For any majority-class sample *x*_*i*_∈*M*, if LOF_*k*_(*x*_*i*_)>*α*, it is identified as a noisy sample and removed from the training set. Minority-class samples are not removed in order to preserve the integrity of the minority-class information.

After LOF-based purification, consisting of minority-class samples *D*_*min*_ and majority-class samples *D*_maj_. On the purified dataset, samples lying near the class boundary are further identified, and majority-class samples with an excessive number of minority-class neighbors are eliminated. For each minority-class sample *x*_*i*_∈*S*, its *k* nearest neighbors in *D*_*min*_ are computed, and the number of majority-class neighbors among them, denoted as *m*_*i*_, is counted. The proportion of majority-class neighbors is then calculated as shown in Equation 8.

(Equation 8) $$\rho_{i}^{maj}=\frac{m_{i}}{k},i=1,...,\left|S\right|$$

Where *m*_*i*_ represents the number of majority-class samples among the *k* nearest neighbors of *x*_*i*_, *k* is the predefined number of nearest neighbors, and |*S*| denotes the total number of minority-class samples. For each sample *x*_*i*_∈*D*_*min*_ belonging to a minority class, after calculating the number of neighbors *ρ*_maj_(*x*_*i*_) belonging to the majority class, the sample criteria are determined using Equation 9.

(Equation 9) $$x_{i}\in \left\{ \begin{array}{cc} D_{\text{border}}, & \text{if}\;0.5<\rho_{\text{maj}}\left(x_{i}\right)<1\\ D_{\text{noise}}, & \text{if}\;\rho_{\text{maj}}\left(x_{i}\right)=1\\ D_{\text{core}}, & \text{if}\;\rho_{\text{maj}}\left(x_{i}\right)\leq 0.5 \end{array}\right.$$

Where *D*_border_ denotes the borderline sample set selected for subsequent oversampling, *D*_noise_ denotes noisy samples to be removed, and *D*_core_ denotes core minority samples that are reliably preserved. The borderline set *D*_border_ serves as the seed set for the subsequent *k*-means clustering and oversampling stages. If the majority proportion satisfies $0.5<\rho_{i}^{\text{maj}}<1$, then *x*_*i*_ is assigned to the borderline sample set *D*_border_. If $\rho_{i}^{\text{maj}}=1$, meaning that the sample is completely surrounded by majority instances, it is regarded as a noisy sample and removed from *D*_*min*_. Similarly, for each remaining majority-class sample *x*_*j*_∈*D*_maj_, the proportion of minority-class neighbors, denoted as $\rho_{j}^{m\text{in}}$, is computed as shown in Equation 10.

(Equation 10) $$\rho_{j}^{m\text{in}}=\frac{m_{j}}{k}$$

Where *m*_*j*_ denotes the number of minority neighbors. If $\rho_{j}^{\min}>0.5$, the corresponding majority-class sample is regarded as potential noise and removed from the training set. On this basis, the borderline sample set *D*_border_ is retained for HCAB-SMOTE oversampling. To prevent excessive deletion of boundary samples, the Minority preservation module enforces two protective constraints. First, the number of majority samples removed by LOF and boundary filtering is capped at 3 % of ∣*D*_maj_∣. This cap ensures that only truly anomalous samples are eliminated, as genuine outliers typically constitute only a small fraction of a dataset; exceeding this proportion would indicate that the detected points are more representative of the data distribution rather than true anomalies. In practice, the LOF algorithm computes outlier factors and removes the top-ranked majority samples until the cap is reached, ensuring that only the most anomalous samples are eliminated. Second, the total number of minority-class samples after all filtering operations shall not fall below 97 % of its original size, which calculates the maximum number of minority samples allowed to be removed using Equation 11.

(Equation 11) $$R_{\min}=\min\left(0.03\times |D_{\min}|\right)$$

Where *R*_*min*_ denote the maximum allowable number of minority samples to be removed, ∣*D*_*min*_∣ represents the total number of original minority class samples. If the boundary filtering step removes a few samples below the threshold, the deleted few samples will be restored in ascending order of their LOF scores, first restoring the sample with the smallest anomaly among the removed samples to retain enough seed samples for oversampling. These two constraints work in tandem to ensure that the subsequent oversampling process operates on a sufficiently large and representative sample pool. The *k*-means clustering algorithm is applied to partition *D*_border_ into *K* clusters by minimizing the within-cluster sum of squares, as shown in Equation 12.

(Equation 12) $$\min_{{\left\{\mu_{t}\right\}}_{t=1}^{K}}\sum_{t=1}^{K}\sum_{x\in C_{t}}∥x-\mu_{t}{∥}^{2}$$

Where *C*_*t*_ denotes the *t*-th cluster, and ***μ***_*t*_ is its centroid. Each sample *x*∈*D*_border_ is assigned to the nearest cluster center as shown in Equation 13. The cluster centers are then iteratively updated according to Equation 14.

(Equation 13) $$cluster\left(x\right)=\arg\min_{t\in \left\{1,\ldots ,K\right\}}∥x-\mu_{t}{∥}^{2}$$

(Equation 14) $$\mu_{t}=\frac{1}{\left|C_{t}\right|}\sum_{x\in C_{t}}x$$

The number of samples in each cluster is computed as *n*_*t*_=∣*C*_*t*_∣. The number of synthetic samples to be generated in each cluster is set equal to the original number of samples in that cluster, i.e., $n_{t}^{\text{syn}}=\lceil \beta \cdot n_{t}\rceil$, where *β* denotes the oversampling ratio that controls the number of newly generated samples per cluster. For each sample *x*_*i*_ in cluster *C*_*t*_, one of its *m* nearest neighbors of the same class is randomly selected as *x*_*j*_, and a new synthetic sample is generated via linear interpolation, as given in Equation 15.

(Equation 15) $$x_{new}=x_{i}+\lambda \cdot \left(x_{j}-x_{i}\right)$$

Where *λ* is a random number uniformly distributed in the interval [0,1]. This process is repeated until $n_{t}^{\text{syn}}$ new samples are generated. To prevent the LOF step from accidentally removing minority samples, all original minority samples are retained after oversampling and merged with the newly generated synthetic samples. If the retention rate of a certain minority class falls below a predefined threshold, additional original samples are supplemented to ensure that the information of that class is not lost. Finally, the synthetic samples are added to the purified training set, resulting in a balanced dataset *D*_balanced_ for subsequent classifier training.

### Quantification and statistical analysis

All analyses were performed in MATLAB R2023. LOF, k-means, SMOTE, Borderline-SMOTE, and HCAB-SMOTE were implemented based on their published algorithms, and the Bagging ensemble used 50 base estimators. All experiments were independently repeated ten times. In this study, *n* refers to the number of segmented vibration signal samples, where each sample contains 4096 points in Case I and 1024 points in Case II; for each fault class, 20 training samples were used, while the normal class contained 140 or 200 samples to form imbalance ratios of 1:7 and 1:10, and the test set contained 100 samples per class. Raw signals were high-pass filtered, time-frequency features were extracted, and PCA was used only for visualization. LOF was applied only to majority-class samples with *α*=2.5, and removal was capped at 3 % of the majority class, while minority-class samples were never removed. Accuracy, precision, recall, F1-score, and G-mean are reported as mean ± standard deviation (SD), and statistical significance was assessed using paired t-tests or Wilcoxon signed-rank tests (*p* <0.05), with normality checked by the Shapiro–Wilk test. All statistical details are explained in the legends and tables of the paper.
